# Preface to the First Edition

**Published:** 1896

**Authors:** Constantine Hering


### PREFACE TO THE FIRST EDITION.

This number is a specimen of the book, which will be published without delay if the profession desires it. The first part of the work will contain full advice: *How the book is to be used.*

The *arrangement*, followed not only through the whole work, but also in its divisions and sub-divisions, will be given separately in the first number, and will be fully explained. The more the reader is familiar with this arrangement, with the more advantage and saving of time he may use the work. The arrangement is the same as has been followed in the monographies of proved drugs in the Journal for Materia Medica, and afterwards in the Hahnemannian Monthly.

A *list of names* will be given of all the medicines mentioned, and their *abridgments;* each will contain two syllables at least, to avoid not only the many errors occurring in nearly all our repertories, as f. i.: *Arn.* and *Ars.; Bell.* and *Hell.; Bar.* and *Bor.; Bor.* and *Bov.; Euph.* and *Euphr.; Sel.* and *Sil.*, etc.; but also to make the reading as easy as possible. It costs more space, but it saves time; and our time is worth more than the few dollars to be given for a more spacious book, if it is an every day's saving for years.

A few alterations in names have been adopted. The old name *Actæa racemosa*, has been, for many reasons, preferred to the later bad one: "Cimicifuga;" the *Calcarea* of Hahnemann has not been called "carbonica," because it is not this chemical combination, but has been named *Calcarea ostrearum*, as a product of an animal, differing widely from the former; for the stale "China," *Cinchona* has been used; otherwise the nomenclature adopted by the Institute has been followed.

A beginning practitioner of Homœopathy cannot have a better opportunity to build up a reputation, than in an epidemic of typhoid character. (Bœnninghausen's Aphorisms of Hippocrates, III. 554, 5.)

A thorough examination of all the elements of each case cannot be too strongly insisted on, or too carefully performed. *In no other disease is this so important, though it is indifferent in none.* (P. P. Wells.)

The totality of the symptoms being once committed to writing, *the most difficult part is accomplished.* (Hahnemann's Organon, § 104.) At the end of his masterly advice: "*How to examine the sick*," Hahnemann also says: After it is done and committed to writing, *the most difficult part is done.*

Beginners may learn by the use of this part of the Analytical Therapeutics, how to complete their images of the single cases, and by summing them up, how to get the totality of symptoms in an epidemic.

If any one should complain of the size of the book, he might be compared to a man, to whom large heaps of bank-notes were offered, and who would refuse to carry them home, because there might be counterfeits among them; saying he found it "too much trouble" to pick them out.

Most of the symptoms are taken from cases reported as cured, or observed by myself, and trustworthy colleagues; only some exceptions have been considered useful, f. i.: The leading symptoms of *Fluor. ac.* have been added, in hope of confirmation by cures. Repetitions of the same words, as used by different physicians, have intentionally not been avoided, but given as near as possible in the words of the observer.

Beginners may be aided by being enabled to glance over in a small compass, all that has already been observed by others, and with success, and thus may by preference, be given again, if the characteristics correspond.

Next to this collection of symptoms, the one for intermittents ought to be used; and next to this, the general repertory of Materia Medica, and above all, Materia Medica itself, because we ought not to make ourselves dependent on former cures. There was a time when *Taraxacum* had never been given in Typhoid, or Intermittent fever, until Bœnninghausen published his case cured, after the *Rhus* had failed, and Raue corroborated it. (Special Path., p. 586.)

This is the right way to widen our circle all around, while strengthening it within.

Corroborated symptoms ought to be marked as such by every practitioner according to his experience; it will, when published, increase their value.

The importance of a medicine in diseases of the same group, or in given symptoms, has, according to Bœnninghausen, been marked; but by signs, instead of different types. The lines | || **I II** indicate their value; the dash (—) refers to the word, or words heading the paragraph, generally printed in bolder type.

At the close of some divisions an index will be found (in this number after the Mind Symptoms), for such as are not used to the rules of the scientific arrangement. This arrangement has the immense advantage of placing similar sensations near each other, instead of the most annoying way of scattering them, according to their first letters, which necessitates a search through the repertory for the word preferred by the author.

Every index refers to the number of the page, and also indicates the column, and the place in the column, where the word is to be found. On the margin of each page are small numbers from one to five. These numbers being placed before, or to the left of the number of the page, indicate the column to the left of the reader; if placed after it, or to its right, they indicate the column to the right of the reader; f. i., 5 **6** refers to page six, left column, part fifth; **6** 5 refers to page six, part fifth, right column.

The arrangement of the *Mind Symptoms* we have to thank Dr. Raue for. The little index added will facilitate the finding of each peculiarity. Every one will find how absurd an alphabetical arrangement would have been.

Dr. P. P. Wells' Treatise on Typhoid Fevers, Am. Hom. Rev., Vol. 3, the best in our literature up to this day, has been included.

H. V. Miller's very serviceable comparison in the Journal of Materia Medica, Vol. V, p. 419, has also been used. Jahr's Forty Years Practice has been frequently quoted verbatim.

The whole work will be published if wanted, and given to the trade, in parts, two dollars and a half each; how many there will be, it is impossible to calculate, but *each one will be complete in itself, and may be used as such by the practitioner.*

Every one who thinks this work may aid our cause, ought to aid the book by sending remarks, and additional observations, which will be thankfully acknowledged by the author.

CONSTANTINE HERING

